# Prediction error and accuracy of intraocular lens power calculation in pediatric patient comparing SRK II and Pediatric IOL Calculator

**DOI:** 10.1186/1471-2415-10-20

**Published:** 2010-08-25

**Authors:** Azlyn-Azwa Jasman, Bakiah Shaharuddin, Raja-Azmi M Noor, Shatriah Ismail, Zulkifli A Ghani, Zunaina Embong

**Affiliations:** 1 Department of Ophthalmology, School of Medical Sciences, Universiti Sains Malaysia, 16150 Kubang Kerian, Kelantan, Malaysia; 2 Department of Ophthalmology, Hospital Raja Perempuan Zainab II, 15000 Kota Bharu, Kelantan, Malaysia

## Abstract

**Background:**

Despite growing number of intraocular lens power calculation formulas, there is no evidence that these formulas have good predictive accuracy in pediatric, whose eyes are still undergoing rapid growth and refractive changes. This study is intended to compare the prediction error and the accuracy of predictability of intraocular lens power calculation in pediatric patients at 3 month post cataract surgery with primary implantation of an intraocular lens using SRK II versus Pediatric IOL Calculator for pediatric intraocular lens calculation. Pediatric IOL Calculator is a modification of SRK II using Holladay algorithm. This program attempts to predict the refraction of a pseudophakic child as he grows, using a Holladay algorithm model. This model is based on refraction measurements of pediatric aphakic eyes. Pediatric IOL Calculator uses computer software for intraocular lens calculation.

**Methods:**

This comparative study consists of 31 eyes (24 patients) that successfully underwent cataract surgery and intraocular lens implantations. All patients were 12 years old and below (range: 4 months to 12 years old). Patients were randomized into 2 groups; SRK II group and Pediatric IOL Calculator group using envelope technique sampling procedure. Intraocular lens power calculations were made using either SRK II or Pediatric IOL Calculator for pediatric intraocular lens calculation based on the printed technique selected for every patient. Thirteen patients were assigned for SRK II group and another 11 patients for Pediatric IOL Calculator group. For SRK II group, the predicted postoperative refraction is based on the patient's axial length and is aimed for emmetropic at the time of surgery. However for Pediatric IOL Calculator group, the predicted postoperative refraction is aimed for emmetropic spherical equivalent at age 2 years old. The postoperative refractive outcome was taken as the spherical equivalent of the refraction at 3 month postoperative follow-up. The data were analysed to compare the mean prediction error and the accuracy of predictability of intraocular lens power calculation between SRK II and Pediatric IOL Calculator.

**Results:**

There were 16 eyes in SRK II group and 15 eyes in Pediatric IOL Calculator group. The mean prediction error in the SRK II group was 1.03 D (SD, 0.69 D) while in Pediatric IOL Calculator group was 1.14 D (SD, 1.19 D). The SRK II group showed lower prediction error of 0.11 D compared to Pediatric IOL Calculator group, but this was not statistically significant (p = 0.74). There were 3 eyes (18.75%) in SRK II group achieved acccurate predictability where the refraction postoperatively was within ± 0.5 D from predicted refraction compared to 7 eyes (46.67%) in the Pediatric IOL Calculator group. However the difference of the accuracy of predictability of postoperative refraction between the two formulas was also not statistically significant (p = 0.097).

**Conclusions:**

The prediction error and the accuracy of predictability of postoperative refraction in pediatric cataract surgery are comparable between SRK II and Pediatric IOL Calculator. The existence of the Pediatric IOL Calculator provides an alternative to the ophthalmologist for intraocular lens calculation in pediatric patients. Relatively small sample size and unequal distribution of patients especially the younger children (less than 3 years) with a short time follow-up (3 months), considering spherical equivalent only.

## Background

Management of childhood blindness is priority in the 'Vision 2020: the right to sight'. Cataract is a major cause of blindness in children throughout the world, particularly in developing countries [1] because of its potential for inhibiting and restricting early visual development.

Early surgery now is universally accepted for younger age children with cataract [2], and the placement of an intraocular lens in children and infants undergoing lens aspiration is gaining wider acceptance [3,4]. However few major issues need to be addressed when determining the power of intraocular lens to be implanted. Should a myopic shift be anticipated in the calculation? And if myopic shift need to be considered, how much, at what age and what is the target refraction should be sought immediately following the implantation?

A wise choice of desired postoperative refraction for the individual patient is crucial in the calculation of intraocular lens power. It is fundamental that the calculation of intraocular lens power should be as accurate as possible in giving a predictable postoperative refraction. The accuracy of this cataract and 'refractive surgery' will permanently enhance the patient's visual life, whereas inaccurate postoperative refractive error may result in lifelong problems.

A number of intraocular lens power calculation formulas have been developed and their accuracy reported [5-7]. There is no general consensus as to which approach or which particular formula is the most accurate.

The Sanders-Retzlaff-Kraff (SRK) power formula, originally derived and published in 1980-1981, has become the most widely used formula for implant power calculation throughout the world [8-10]. However, we must bear in our mind that this formula does not consider myopic shift, one of the important element in calculating intraocular lens power in pediatric age group.

All children undergo a myopic shift. In normal eyes of children, axial length increases rapidly until 2 to 3 years of age, slow and stabilizes between 8 and 10 years of age. In contrast, corneal curvature decreases with age and stabilizes at approximately 1 year of age [11].

Because of the complexity of the functions of the eye and the numerous factors involved in its refraction, the calculation of the implant power is somewhat complicated. Axial elongation and changes in corneal curvature are major factors influencing refractive changes in the early childhood life. It is thought that the presence of cataract, surgical removal of cataract and the implantation of an intraocular lens into the eye; influence the further growth of the eye, thus create difficulties regarding the choice of the power of the appropriate intraocular lens [12].

Pediatric Intra-ocular Lens (IOL) Calculator is a modification of SRK II using Holladay algorithm. This program attempts to predict the refraction of a pseudophakic child as he grows, using a Holladay algorithm model. This model is based on refraction measurements of pediatric aphakic eyes. Pediatric IOL Calculator uses computer software for intraocular lens calculation.

**Figure 1:**
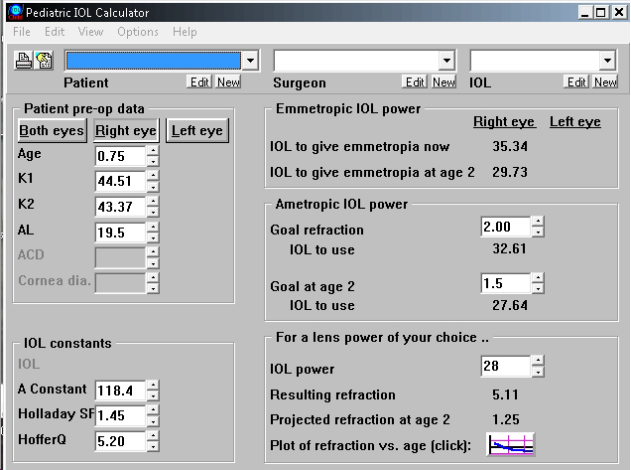
**A screen capture of the Pediatric IOL Calculator**.

The Pediatric IOL Calculator for pediatric intra-ocular lens calculation comes with a program. The model used in this program is based on analysis of the refractive changes in aphakic children who underwent surgery before the age of 10 years (with documented refractions for more than 7 years) and it is collaborated with the predictions of a logarithmic model of myopic shift [13,14]. This program calculates the predicted refraction of a child made pseudophakic, given biometric measurements and intraocular lens parameters. It shows this prediction in graphical form, and allows the surgeon to dynamically view the effects of changing any parameter. It also allows the surgeon to see how closely the actual refractions match those predicted by the program (Figure 1). It is not valid for ages less than 3 months (0.25 years) as the program does no calculations for ages younger than 3 months. For premature children, the corrected gestational should be used in place of the actual age.

This study is designed to compare the mean prediction error and the accuracy of predictability of the intraocular lens power calculation in pediatric patients after cataract surgery with primary implantation of intraocular lens using SRK II versus Pediatric IOL Calculator for pediatric IOL calculation.

## Methods

### Subjects

A comparative study was conducted from August 2005 to May 2008, at Hospital Universiti Sains Malaysia and Hospital Raja Perempuan Zainab II, Kelantan, Malaysia.

The sample size was calculated using 'Power and Sample Size' software, version 2.1.25. Based on the research design and strategy, sample size was calculated using Two Means Formula with 90% power of study. The minimum requirement for each group was 14 patients.

We include all pediatric patients with visually significant, dense cataract (congenital and developmental cataract), age between 3 months and 12 years old at time of intraocular lens implantation and patients with cornea abnormality, persistent hyperplastic primary vitreous, history of ocular surgery, history of ocular trauma and post cryotherapy were excluded from the study.

### Sampling procedure

Simple randomize sampling was used. In this process, envelope technique sampling procedure was conducted. A stack of opaque sealed envelope was prepared with 14 envelopes containing a piece of paper with the word 'SRK II' and the remaining 14 envelopes stated 'Pediatric IOL Calculator'. The Primary Investigator would draw an envelope for each patient during the preoperative day. The intraocular lens power then calculated based on the printed technique selected for every patient either SRK II or Pediatric IOL Calculator for pediatric IOL calculation.

### Definition of terms

#### 1. Predicted refraction

Predicted refraction is the target postoperative refraction after cataract operation. Predicted refraction value derived from the intraocular lens implant formula. The predicted postoperative refraction is not always emmetropia depending on the patient's age and surgeon preference.

#### 2. Observed refraction

Observed or actual refraction is the refractive value expressed in spherical equivalent seen in the patient postoperatively. The refraction is taken as the final value after stable refraction. Observed refraction values derived from the cycloplegic refraction.

#### 3. Spherical equivalent

Spherical equivalent is defined as average spherical power of the eye or spherocylindrical lens for a given period. It is calculated by the following equation [15].

Spherical equivalent=sphere+½ cylinder

#### 4. Prediction error

The prediction error is the absolute difference between the predicted and the observed refraction. This is expressed in spherical equivalent (diopter) [16,17].

#### 5. Predictability

Patient's postoperative refraction is subtracted from the predicted refraction (in spherical equivalent). For this study, eyes with postoperative refraction within ± 0.5 D of expected refraction are considered accurate or good predictability, while those with final refraction more than ± 0.5 D from predicted refraction is considered inaccurate, reflecting poor predictability.

### Study procedure

#### 1. Preoperative assessment

After informed consent was taken, all potential patients were randomized into 2 groups; for SRK II group and Pediatric IOL Calculator group. The children in both groups were then underwent a complete ocular assessment in both centres (Hospital Universiti Sains Malaysia and Hospital Raja Perempuan Zainab II). Clinical data concerning patient's demography was documented. Visual acuity of both eyes was tested for children who cooperative using standard retro illuminated Snellen chart or Sheriden Gardiner test and the best corrected visual acuity was documented. Slit lamp examination was done in all subject either using the normal slit lamp biomicroscope or a portable slit lamp.

A contact method A-scan biometry, Quantel Medical Axis-II PR was used in this study to determine the axial length of the eyeball. The axial length is the distance between the anterior surface of the cornea and the fovea. The patient was asked to sit on the chair and look straight ahead. In case of general anesthesia, axial length was measured without foveal fixation. The probe was placed against the cornea with minimum pressure as light as possible. The average of five measurements was used. The axial length is measured in millimetre (mm).

The Humphrey 599 autokeratometer is used to measure the cornea power. The patient sat comfortably on the patient side of the unit. The patient's canthus was ensure at the same level as the marker on the side of the forehead rest assembly, while the patient's forehead rested comfortably against the headpiece. The patient then was asked to hold still and look at the target. The instrument tracking function would begin automatically. The instrument then roughly aligned using the joystick, so that the patient's pupil was located within the white box on the screen. Once the pupil is inside the white box, the automatic alignment took over and completed the alignment process. The cornea radius curvature is measured in millimetre (mm) and converted to diopter (D). In a case of keratometry done under sedation or general anaesthesia, the Nikon Retinomax K-Plus-2 autorefractokeratometer was used.

Axial length and keratometry measurements were done under sedation or general anaesthesia in children unable to follow the instruction and in younger children (less than 3 years old).

#### 2. Intraocular lens power calculation and selection

The power of the intraocular lens implant was calculated using SRK II or Pediatric IOL Calculator as assigned earlier based on the axial length and keratometry.

Instead of the original SRK regression formula states that;

P = A − 2.5 L − 0.9 K

where

P = emmetropia intraocular lens power in dioptres (D)

A = a constant which reflects the position of the particular model of implant within the eye

L = axial length in millimetre (mm)

K = average keratometry in dioptre (D)

the SRK II formula has been revised by Sanders [8] in order to alter the A constant depending on axial length. The A constant is adjusted (and termed as A1) in a step-wise way over the range of axial lengths.

axial length < 20 mm; A1 = A + 3

20.00 mm ≥ axial length < 21.00 mm; A1 = A + 2

21.00 mm ≥ axial length < 22.00 mm; A1 = A + 1

22.00 mm ≥ axial length < 24.50 mm; A1 = A

Axial length ≥ 24.5 mm; A1 = A - 0.5

In this study, the predicted postoperative refraction for SRK II group is aimed for emmetropic at the time of surgery.

Pediatric IOL Calculator is a modification of SRK II using Holladay algorithm. This program attempts to predict the refraction of a pseudophakic child as he grows, using a Holladay algorithm model. This model is based on refraction measurements of pediatric aphakic eyes. Pediatric IOL Calculator uses computer software for intraocular lens calculation. In this study, the predicted postoperative refraction for Pediatric IOL Calculator group is aimed for emmetropic spherical equivalent at age 2 years old.

The lens power and estimated postoperative refraction for both groups were recorded.

#### 3. Surgical procedure

The surgeries had been performed by a single surgeon in each centre. Preoperatively, the pupillary dilatation was accomplished with cyclopentolate 1%. This was done at least one to two hours before the surgery to ensure optimum pupil dilatation.

Standard lens aspiration surgeries were performed in all patients. Limbal scleral-tunnel incision was made at 12 o'clock using 2.75 mm knife. A small paracentesis was placed 45°away from the sclera incision. Viscoelastic device is injected into the anterior chamber to maintain the anterior chamber depth. A cystitome is introduced into the anterior chamber and a small radial cut is made in the anterior capsule and followed with a continuous curvilinear capsulorhexis by using an utrata forcep. Hydrodisection done and followed by irrigation and aspiration using simcoe to remove the lens material. Then the anterior chamber and capsular bag were expanded with the viscoelastic device.

Primary posterior capsulotomy was made followed by limited anterior vitrectomy. A foldable intraocular lens was then implanted in the capsular bag. The foldable intraocular lens that was used in this study is AcrySof SN60AT (Alcon lab). All patients were implanted with the same type of intraocular lens in order to standardize intraocular lens in both groups. The viscoelastic material was removed using the irrigation/aspiration simcoe. The incision was sutured with 10/0 nylon. Subconjunctiva injection of dexamethsone 0.5 mg/0.125 ml combined with gentamicin 10 mg/0.25 ml was given before the operated eye being padded with ointment chloramphenicol.

#### 4. Postoperative treatment, follow-up and refraction

Postoperatively, all patients in both groups were prescribed syrup acetazolomide 125 mg three times daily for a day. After being reviewed on day one postoperatively, they were discharged home with gutt prednisolone acetate 1% two hourly for a week, tapered to four hourly for a month and six hourly for another three months. They were also given chloramphenicol and cycloplegic ophthalmic solution.

All patients were reviewed regularly (at one week, one month and three months postoperatively) and given same medication regime. A subjective refraction was done at 3 months postoperatively. Three month was chosen as the wound is considered to be completely healed and stable.

Welch-Allyn retinoscope was used to objectively determine the spherocylindrical refractive error. The refraction set used in our study was Meniscus Trial Lenses MSD, 13/15 21052 Massari Mazoti. Cyclopentolate hydrochloride 1% was used for pupillary dilatation and cycloplegia prior to refraction with streak retinoscope. The patient was asked to sit on a chair and look straight ahead and focus on an eye chart about 20 feet away. The refraction proccedure is performed by an optometrist. Objective refraction was done in all patients and subjective refraction was only done in children who have sufficient language skills. We found that the result for both subjective refraction and objective refraction were almost similar. The subjective refraction is used as the end result of postoperative refraction and is converted into spherical equivalent expressed in diopter (D). For children who still did not have sufficient language skills to undertake subjective refraction, objective refraction was used as the end result of postoperative refraction. The objective refraction was done using Nikon Retinomax K-Plus 2 autorefractokeratometer under sedation in children who unable to follow the instruction and in younger children (less than 3 years old).

### Statistical analysis

Comparison was made later between the two groups of different formula; children underwent lens aspiration with intraocular lens power calculated using SRK II and Pediatric IOL Calculator for pediatric IOL calculation. The comparison focuses on cases in which those formulas accurately predict the postoperative refraction to within ± 0.5 D (as the intraocular lens comes in 0.5 D step) and cases in which those two formulas do not accurately predict postoperative refraction, which differs by more than ± 0.5 D from the actual target refraction. The data collected were analyzed using Statistical Package for Social Science (SPSS) software version 12.1.

### Plans for minimizing study error

The following steps were taken to reduce possible errors while performing this study;

i. Only patients whom met the inclusion and exclusion criteria were selected

ii. All preoperative biometry parameters were measured by two persons for every single patient to get average reading and avoid observer bias in each centre

iii. The average of at least five measurements of axial length with least standard deviation was taken as a value

iv. The same instruments were used for measurement in each centre

v. All patients were given similar regime of treatment and follow-up

This study was approved by the Research and Ethical Committee, School of Medical Sciences, Universiti Sains Malaysia.

## Results

### Demographic data

A total of 24 patients with 31 eyes were studied over a period between August 2005 and May 2008. Thirteen patients (16 eyes) were assigned for SRK II group and another 11 patients (15 eyes) for Pediatric IOL Calculator group.

For the overall group of 31 eyes, mean age of patients underwent lens aspiration and intraocular lens implantation was 6.84 years (SD, 3.42 years) with the minimum age was 4 months and maximum age was 12.25 years old. In relation to the SRK II group, the mean age at surgery was 7.21 years (SD, 3.22 years) with a minimum and maximum age of 1.17 and 12.17 years old respectively. In the Pediatric IOL Calculator group, the mean age was 6.45 years (SD, 3.68 years) with a minimum age of 4 months and maximum of 12.25 years old.

There were 8 boys (61.54%) and 5 girls (38.46%) in the SRK II group while 5 boys (45.45%) and 6 girls (54.55%) in the Pediatric IOL Calculator group as shown in Table 1. Table 1 also showed the distribution of ethnicity for both groups.

**Table 1:** Distribution of patients according to gender and ethnic group

	SRK II	Pediatric IOL Calculator
	n = 11	n = 13
	n (%)	n (%)
Gender		
Boy	8 (61.54%)	5 (38.46%)
Girl	5 (45.45%)	6 (54.55%)

Ethnic		
Malay	12 (92.31%)	1 (7.69%)
Chinese	1 (7.69%)	1 (9.09%)
Indian	0	1 (9.09%)

Out of 24 patients, 14 patients had bilateral cataract and another 10 patients had unilateral cataract. However, from those 14 patients with bilateral cataract, only 7 patients underwent bilateral lens aspiration and intraocular lens implantation. The remaining 7 patients did only one eye operation as the cataract of the fellow eye was not visually significant.

### Distribution of eyes according to age, axial length and keratometry at time of surgery

#### a) Age

The distribution of eyes according to age at the time of surgery in each group is shown in Table 2. Eighty seven percent of our patients were equal or older than three years old at time of surgery.

**Table 2:** Distribution of eyes according to age at time of surgery

	Number of Eyes		Total of Eyes
Age at surgery	SRK II	Pediatric IOL Calculator	n(%)
< 3 years old	2	2	4 (13%)
≥ 3 years old	14	13	27 (87%)

#### b) Axial length

**Figure 2:**
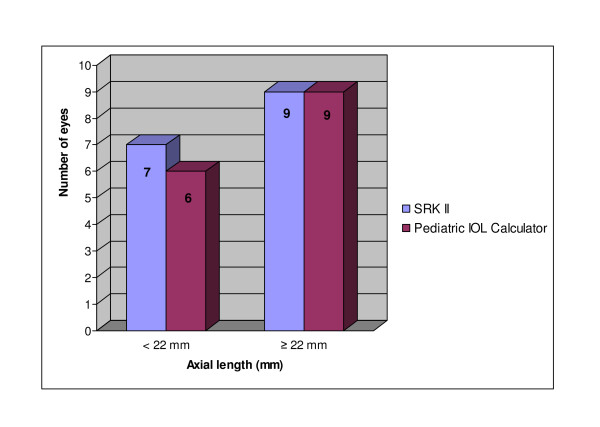
**Distribution of axial lengths in both groups in pediatric population**.

The axial length measurements in both groups are detailed in Figure 2. Fifty-eight percent (18 eyes) out of 31 eyes had axial length equal or more than 22 mm. The remaining had axial length of less than 22 mm. Table 3 showed the mean axial length in both groups. There was no statistically significant difference of axial length measurement between the two groups (p = 0.75).

**Table 3:** Mean axial length and keratometry in both groups

		Overall	SRK II	Pediatric IOL Calculator	*p value
Axial length (mm)	[mean (SD)]	22.51 (1.88)	22.61 (1.86)	22.39 (1.96)	0.75
	[range]	18.93 - 27.04	20.16 - 27.04	18.93 - 26.60	

Keratometry (diopter)	[mean (SD)]	44.22 (2.60)	44.71 (2.69)	43.70 (2.48)	0.28
	[range]	37.01 - 50.13	40.06 - 50.13	37.01 - 46.37	

* Independent-Samples T Test

p value < 0.05 (significant)

#### c) Keratometry

**Figure 3:**
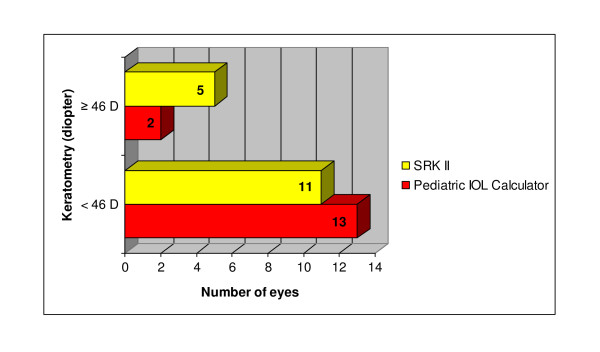
**Distribution of keratometry in both groups in pediatric population**.

Keratometry reading was classified into two subgroups; less than 46 D and equal or more than 46 D. Majority of our patients had keratometry of less than 46 D in which 11 eyes in SRK II group and 13 eyes in Pediatric IOL Calculator group (Figure 3).

For the overall group of 31 eyes, mean keratometry was 44.22 D (SD, 2.60 D) with the range from 37.01 D to 50.13 D. The mean keratometry for SRK II and Pediatric IOL Calculator group were 44.71 D (SD, 2.69 D) and 43.70 D (SD, 2.48 D) respectively. There was no statistically significant difference of keratometry measurement between patients receiving intraocular lens power calculated using SRK II or Pediatric IOL Calculator (p = 0.28) (Table 3).

### Comparison of prediction error between SRK II and Pediatric IOL Calculator

The mean prediction error in the SRK II group was 1.03 D (SD, 0.69 D) while in Pediatric IOL Calculator group was 1.14 D (SD, 1.19 D). Though the SRK II group shows lower prediction error of 0.11 D compared to Pediatric IOL Calculator group, but this was not statistically significant (p = 0.74) (Table 4).

**Table 4:** The mean predicted refraction, observed refraction and prediction error between SRK II and Pediatric IOL Calculator

	SRK II	Pediatric IOL Calculator	*p value
Mean Predicted Refraction (Diopter, SD)	-0.06 (1.17)	1.19 (2.35)	
Mean Observed Refraction (Diopter, SD)	-0.84 (1.31)	0.10 (1.74)	
Mean Prediction Error (Diopter, SD)	1.03 (0.69)	1.14 (1.19)	0.74

* Independent-Samples T Test

p value < 0.05 (significant)

The data were subdivided for eyes with age at surgery of less than 3 years old, and equal or older than 3 years old; axial lengths of less than 22 mm, and equal or more than 22 mm; and keratometry measurement of less than 46 D, and equal or more than 46 D. Table 5 shows the mean prediction error for the subgroups.

**Table 5:** Mean prediction errors for the subgroup data

	Mean Prediction Error (Diopter, SD)		
	SRK II	Pediatric IOL Calculator	* p value
Age			
Age < 3 years	1.55 (1.46)	3.54 (0.33)	0.201
Age ≥ 3 years	0.94 (0.57)	0.77 (0.74)	0.488
Axial length			
Axial length < 22 mm	0.91 (0.77)	1.47 (1.65)	0.439
Axial length ≥ 22 mm	1.11 (0.64)	0.91 (0.80)	0.580
Keratometry			
Keratometry < 46D	0.93 (0.61)	1.20 (1.26)	0.525
Keratometry ≥ 46D	1.22 (0.86)	0.72 (0.65)	0.499

* Independent-Samples T Test

p value < 0.05 (significant)

The mean prediction error was greater in eyes in children less than 3 years old at time of surgery. There was a trend toward a smaller prediction error in eyes in children equal or older than 3 years old. However, there was no statistically significant difference in both groups (p = 0.488).

In term of axial length and keratometry, a trend toward a smaller prediction error was observed in eyes with longer axial length and larger keratometry in the Pediatric IOL Calculator group. Conversely, SRK II group showed the longer the axial length and the larger the keratometry, the prediction errors became greater. All these findings were comparable between the two groups.

### Comparison of the accuracy of predictability between SRK II and Pediatric IOL Calculator

There were 3 (18.75%) eyes in SRK II group where the refraction postoperatively was within ± 0.5 D from predicted refraction compared to 7 (46.67%) eyes in the Pediatric IOL Calculator group. However, there was no statistically significant difference in both groups (p = 0.097).

Further distribution of postoperative refraction is shown in Table 6. Both groups were comparable in achieving postoperative refraction within ± 2.0 D of predicted refraction which was 87.50% and 80.00% of eyes in SRK II and Pediatric IOL Calculator respectively.

**Table 6:** Accuracy of predictability between SRK II and Pediatric IOL Calculator

Observed refraction (*Spherical Equivalent*)	SRK II (n = 16)	Pediatric IOL Calculator (n = 15)	Overall (n = 31)	* p value
≤ ± 2.0 Diopter				
*Accurate*				
0.0 D to ± 0.5 D	3 (18.75%)	7 (46.67%)	10 (32.26%)	0.097
*Inaccurate*				
> ± 0.5 D to ± 1.0 D	7 (43.75%)	0 (0.00%)	7 (22.58%)	
> ± 1.0 D to ± 2.0 D	4 (25.00%)	5 (33.33%)	9 (29.03%)	
> ± 2.0 Diopter	2 (12.50%)	3 (20.00%)	5 (16.13%)	

* Pearson chi-square test

p < 0.05 (significant)

## Discussion

This study is intended to assess the predictability of desired refractive outcomes in the immediate postoperative period in pediatric patients with cataract undergoing lens aspiration with primary intraocular lens implantation.

In our study of 24 patients over 3 years period, a preponderance of the children was boys, (54.16%). This is consistent with the 51.5% to 72.0% of congenital and developmental cataract reported in the literature [2,18,19].

Our data also showed that the distribution of age at time of surgery were more towards older children. Only 4 eyes in children of less than 3 years old were enrolled in our study. This reflects the parental awareness is poor with regards to the diagnosis, early surgical intervention and the need for earlier visual rehabilitation.

A study by Teresa et al [20] of 138 patients has similar findings. The mean age at time of surgery was 3.1 (3.7) years (range 1 week to 20.5 years). In their paper, they did not precisely state the age distribution of their study patients. In a retrospective review by Daniel et al [17] of 101 eyes over 5-year period from June 1998 to August 2003, the mean age at time of surgery was 5.7 (4.4) years old. Their patients' age ranged from 22 days to 18 years old. Their findings compared favourably to us.

### Prediction error

The mean prediction error of the refractive outcome obtained in our study was 1.03 D (SD, 0.69 D) for SRK II group and 1.14 D (SD, 1.19 D) in Pediatric IOL Calculator group. This showed both groups ended more myopic than anticipated. However, there was no statistically significant different in the mean prediction error in both groups. Even though, the SRK II group had a lower prediction error of 0.11 D compared to the Pediatric IOL Calculator, we could not prove that SRK II is better than Pediatric IOL Calculator or vice versa. Our results showed that for the overall group, the prediction error is satisfactory and is comparable with errors demonstrated in adult group [21].

In our study, we further divided and analyzed the prediction error according to age group, axial lengths and keratometry. We divided the groups according to the age at time of surgery to less than 3 years old, and equal or older than 3 years old; axial length of less than 22 mm, and equal or more than 22 mm; and keratometry of less than 46 D and equal or more than 46 D. This was based on the fact that the surgical predictions of appropriate implant power become increasingly complicated in children under age 3 years, especially those under 1 year of age [17,22].

Although our sample size was too small to reach significance level, there was a trend towards a smaller prediction error in eyes in children equal or older than 3 years old in both formulas. The trends also observed in eyes with axial length equal or more than 22 mm and keratometry equal or more than 46 D (which demonstrated in Pediatric IOL Calculator group). Majority of our patients were equal or older than 3 years at the time of surgery. Only 13% (4 eyes) were less than 3 years old. As most of our patients were equal or older than 3 years old, the eyes also had relatively normal axial length.

We postulated that if we were to get equal representation of samples in both age groups, we would be able to prove the benefit of the Pediatric IOL Calculator. It is well known that the rapid growth of the eye in children, especially in the first year of life, increased tissue reactivity, decreased scleral rigidity and alteration in growth patterns of pseudophakic eyes are the major issue in pediatric cataract surgery [13,14,17,23]. These can lead to postoperative surprise.

Tromans et al [24] did a fairly similar study like us. They showed a similar trend of prediction errors. Our mean prediction errors for both groups were also comparable to them. They did a study using SRK II and SRK/T to determine the accuracy of intraocular lens power calculation in a group of 52 pseudophakic eyes of 40 infants and children. Their interest was on the prediction error at three months post operatively in different axial lengths and age.

In their study they divided the groups according to axial length, keratometry reading and also age at surgery. For the overall group the mean prediction error was 1.40 D (SD, 1.60 D). The mean prediction errors in eye with axial length less than 20 mm was 2.63 D (SD, 2.65 D), and in eyes 20 mm or more was 1.07 D (SD, 0.98D). The mean prediction errors in eyes in children aged 36 months or more was 1.06 D (SD, 1.02 D), while patients less than 36 months was 2.56 D (SD, 2.50 D). The differences between the prediction errors for both axial length and age were statistically significant (p < 0.05).

### Accuracy of predictability

In term of accuracy of the predictability in our study, both formulas did not show any significant difference. The Pediatric IOL Calculator group accurately predicted the immediate postoperative refraction within ±2 D in 80% of patient; while the SRK II group in 87.5% of patients. A study by McClatchey [13,14] using this Pediatric IOL Calculator found that this formula accurately predicted the refraction within 3 D in 24% of eyes operated before two years old, and in 77% of eyes operated after this age. The accuracy criterion in our study was very small in which we set the cut point of ± 0.5 D as accurate.

The accuracy of postoperative refraction in this study is comparable with studies done by Daniel et al [17] in which 77% achieved ± 2.0 D. In fact our study showed a better result with 62.5% of eyes achieved ± 1.0 D of prediction error in SRK II group and 46.67% in Pediatric IOL Calculator group.

Although it is difficult to compare individual studies because of the variations between the inclusion criteria, exclusion criteria, certain general conclusion can be drawn. Most of the authors agree that pseudophakic eyes grow normally and a significant shift after intraocular lens implantation is to be expected. The resulting myopic shift would lower the estimated refraction, and this should be borne in mind when comparing estimated and actual refractive outcomes [24].

However, from our study there was no significant difference between SRK II and Pediatric IOL Calculator which showed Pediatric IOL Calculator with its myopic shift element did not outperform the SRK II. Even though McClatchey et al [13,14] with their Pediatric IOL Calculator tried to consider myopic shift as one of important element in the intraocular lens power calculation, still we were unable to obtain a favourable and significant result of predictability in our study. The relatively small sample size and unequal distribution for the group of less than 3 years old could be the explanation for our results.

More information is needed about the growth pattern of the eyes following cataract removal and intraocular lens implantation. With a better understanding of the factors influencing pediatric eye growth will assist in intraocular lens power calculation and the prediction of refractive changes after intraocular lens implantation. This also will increase the confidence of the surgeon in choosing the optimum power of the intraocular lens and most important a better visual quality for the child.

To the best of our knowledge, we are the first to report the predictability of post operative refraction between SRK II and Pediatric IOL Calculator. Though we are unable to get a significant difference between the two formulas, we believe if we were to have a larger sample with longer study duration, we will have more favourable and significant result.

These findings emphasize the differences between adult and pediatric cataract surgery and lend support to arguments for development of a new intraocular lens power calculation formula that addresses the specific needs of the pediatric population. The question of appropriate intraocular lens power selection will require many more years of follow-up in a large number of infants and children before enough data is accumulated to accurately predict the expected refractive change during the rapid growth period.

## Conclusions

The prediction error and the accuracy of predictability of postoperative refraction in pediatric cataract surgery are comparable between SRK II and Pediatric IOL Calculator. This could be explained by relatively small sample size and unequal distribution of patients especially the younger children (less than 3 years) with a short time follow-up (3 months), considering spherical equivalent only. The existence of the Pediatric IOL Calculator provides an alternative to the ophthalmologist for intraocular lens calculation in pediatric patients.

## Competing interests

This study was supported by a short term grant (304/PPSP/6131475) from Universiti Sains Malaysia.

## Authors' contributions

AAJ designed the study protocol, data collection and analysis. BS designed the study protocol and data analysis. RAMN designed the study protocol, performed the surgery and the lens power calculation. ZAG performed the surgery and the lens power calculation. SI involved in the analysis of the study. ZE designed the study protocol, analysis and management of the study. All authors read and approved the final manuscript.

## Pre-publication history

The pre-publication history for this paper can be accessed here:

http://www.biomedcentral.com/1471-2415/10/20/prepub
